# Obsessive-Compulsive Disorder and Suicidality: A Case Control Study

**DOI:** 10.1192/bjo.2024.80

**Published:** 2024-08-01

**Authors:** Swapnil Yadav, V Senthil Kumar Reddi, Jaisoorya Sekharan

**Affiliations:** 1 University Hospital of North Tees, Stockton on Tees, United Kingdom; 2 National Institute of Mental Health and Neurosciences, Bangalore, India

## Abstract

**Aims:**

This study aims to explore the characteristics of the individuals who engage in suicidal behaviour.

**Hypothesis:**

1. Patients attempting suicide are more likely to have co-existing axis I or axis II disorder when compared with patients with no reported suicidal attempt.
2. Various OCD related domain like symptom types, symptom severity, age of onset of OCD, duration of illness and presence of other OCD spectrum disorder has higher chances of attempting suicide.
3. Family history of suicidal behaviour increases the risk of suicidality.

**Methods:**

Retrospective file review of all patients registered at the OCD clinic, NIMHANS hospital, Bangalore, India between Jan 2008–Dec 2018 was undertaken. Out of 1017, 814 met the eligibility criteria. Individuals with a documented suicide attempt were compared with those without. Chi square test, unpaired t-test and Regression analysis was done to identify predictors of life-time attempt.

**Results:**

Lifetime attempt was noted in 19.8% patients (161 out of 814). On comparison, female gender, unemployment, lower socioeconomic status, severe to extreme avoidance, severe to most severe CGIs, presence of depressive disorder, history of engagement in suicidal acts, past NSSI, past suicidal ideation, younger age at onset of OCD, younger age at first OCD consultation and YBOCS at index assessment are significantly associated with higher risk of suicidal attempts. Female gender, BPL status, age at onset of OCD and presence of depressive disorder can significantly predict lifetime suicidal attempts. Out of 814 eligible patients reviewed, 32 patients i.e. 4.79% had made ≥1 suicide attempt after their first contact to the OCD clinic. Risk of re-attempting suicide is highest in the first three years post index visit to the OCD clinic.

**Conclusion:**

One in five individuals with OCD attempt suicide with higher risk in female population, greater illness severity (higher baseline YBOCS scores and early age of OCD onset) and presence of comorbid depression. Importantly, risk of repeated attempt is greatest within three years of contact but no factor could determine reattempt risk. Hence, regular screening for suicidality in patients with OCD could be of utmost importance in preventing any future attempts. The findings also highlight the need for future studies that explore the neurobiological underpinnings of suicide vulnerability in OCD.

